# “Learning, Thinking & Conveying” training with experiential learning in the emergency department for undergraduate nursing students: A quasi-experimental study

**DOI:** 10.1097/MD.0000000000043231

**Published:** 2025-07-11

**Authors:** Ying Li, Shuang Cheng, Di Liu, Wei Jiang

**Affiliations:** a Emergency, The Central Hospital of Wuhan, Tongji Medical College, Huazhong University of Science and Technology, Wuhan, China; b Nephrology, The Central Hospital of Wuhan, Tongji Medical College, Huazhong University of Science and Technology, Wuhan, China.

**Keywords:** critical thinking, emergency nursing, experiential learning, internship experience, nursing students, self-directed learning

## Abstract

**Background:**

“Learning, Thinking & Conveying” training with experiential learning was evaluated for its impact on the theoretical knowledge and clinical operation skills of undergraduate nursing students in the Emergency Department. This was a quasi-experimental study.

**Methods:**

A total of 90 undergraduate nursing students in the emergency department of a tertiary care hospital in Wuhan were assigned to experimental and control groups between July and December 2023. The experimental group received “Learning, Thinking & Conveying’’ training and controls were taught using standard cases. Students completed a questionnaire based on The Critical Thinking Disposition Inventory-Chinese Version (CTDI-CV) and The Self-Rating Scale for Self-Directed Learning (SRSSDL) before and after the experimental period. Theoretical and clinical skills were assessed at the internship end for all participants.

**Results:**

Students in the experimental group showed significant improvement in theoretical performance compared with controls (*P* < .001) but no differences were found in clinical operation skills. Similar CTDI-CV and SRSSDL scores for self-directed learning and critical thinking were found for both groups at the outset and the experimental group showed greater improvement in 5 out of the 7 dimensions of CTDI-CV and every dimension of SRSSDL after 4 weeks.

**Conclusions:**

“Learning, Thinking & Conveying” training in combination with experiential learning during the rotation period improves theoretical performance, self-directed learning and critical thinking in undergraduate nursing students.

## 1. Introduction

Nursing students are vital for the healthcare service and their proficiency has a profound impact on quality of care. The internship phase involves patient interaction and prepares students for clinical practice during which they must apply theoretical knowledge in real-world settings.^[1]^ Emergency departments represent a challenging and high-pressure environment for both students and instructors and traditional teaching methods require continuous monitoring and updating to ensure students acquire the necessary first-aid skills.

**Figure 1.**
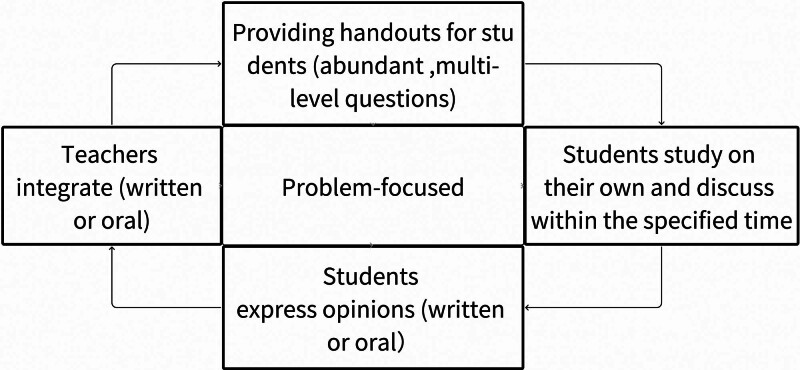
“Learning, Thinking & Conveying” Framework Diagram. Schematic diagram of “Learning, Thinking & Conveying” pedagogy proposed by Zhang Huicheng, Taiwan, China in 2013. “Self-studying,” “thinking,” and “conveying” are emphasized as core components of the learning process. Problem-solving through student group collaboration is encouraged, fostering a classroom environment characterized by cooperation and competition. The aim is to motivate students by focusing on independent learning and analytical thinking. The pedagogy follows a five-step recursive process: self-studying, thinking, discussing, conveying and integrating, promoting continuous engagement and enhancing learning.

“Learning, Thinking & Conveying” pedagogy was proposed by Zhang Huicheng, Taiwan, China in 2013 and emphasizes “self-studying,” “thinking” and “conveying.” Problem-solving is undertaken by student groups to shift the classroom dynamic to one of cooperation and competition. The aim is to motivate students with an emphasis on independent learning and analytical thinking. “Learning, Thinking & Conveying” pedagogy consists of 5 steps: self-studying, thinking, discussing, conveying, and integrating during a recursive process.^[2,3]^ A “Learning, Thinking & Conveying” Framework Diagram to describe learning, thinking conveying is shown in Fig. 1.

Experiential learning includes acting as a model in technique demonstrations and engaging in peer-assisted small-group practice. Students must be actively engaged and experiential learning should mimic real-life situations, fostering curiosity and self-directed learning. This approach has been widely adopted by nursing curricula and allows the practice of procedures and the involvement of patients. Cheng study^[4]^ demonstrated a positive impact of experiential learning on critical thinking skills perhaps due to the internalization of knowledge through cooperation, reflection, questioning, and action.^[5]^ Halpern defined critical thinking as the use of cognitive skills or strategies to increase the likelihood of a desired outcome and such skills aid the making of clinical judgments.^[6]^ The disposition towards critical thinking indicates an individual’s inclination to think critically when problem-solving and is a key competency contributing to the optimization of patient outcomes.^[7,8]^ The enhancement of critical thinking is a primary objective in nursing education.

Self-directed learning (SDL) skills allow nursing students to adapt to the rapidly evolving healthcare situation^[9]^ and promote personal and professional development.^[10]^ Studies on SDL, involving group projects and web-based learning, have been conducted and the present work aims to evaluate the contribution of experiential learning.

Recent studies have demonstrated promising outcomes for experiential learning. The present work introduces a blended teaching model, highlighting “Learning, Thinking & Conveying” training combined with experiential learning. It aims to stimulate SDL and critical thinking and contribute to educational programs for nursing students.

## 2. Methods

### 2.1. Study participants

**Figure 2.**
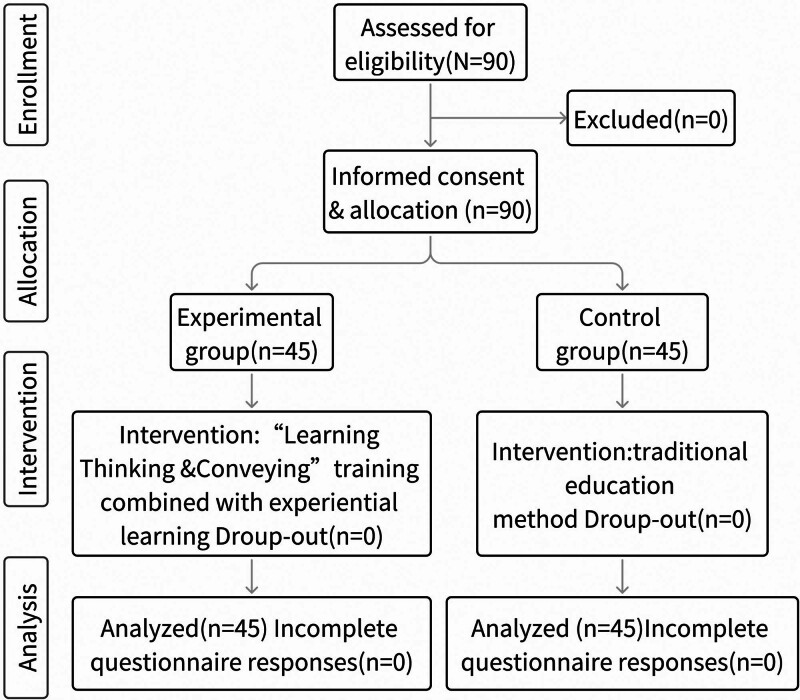
Nursing students flow diagram of emergency department training. Flow diagram visually representing the assignment and progression of nursing students through a 4-week internship in the emergency department (ED) of a tertiary care hospital in Wuhan from July to December 2023. Students were divided into 2 groups: an experimental group who rotated in odd months and a control who rotated in even months.

Nursing students completing a 4-week internship in the emergency department of a tertiary care hospital in Wuhan between July and December 2023 were enrolled and were subdivided into an experimental group of students who rotated in odd months and a control group who rotated in even months. A quasi-experimental design was chosen to maintain the integrity of cohorts of students and thus avoid the confounding influence of dividing previously established groups to receive different training. A research flow diagram is shown in Fig. 2.

Inclusion criteria were as follows: (1) participants were full-time undergraduate nursing students beginning their clinical practice; (2) participation was voluntary and informed consent was given by all participants. Exclusion criteria included: (1) taking leave for more than 3 consecutive days during the training period; (2) having incomplete training or assessment records; (3) being unable to complete the clinical practice for personal reasons. Ethical approval was granted by the ethics review board of our hospital.

The sample size was calculated using G*Power 3.1 software based on the formula for 2 independent groups: n1 = n2 = 2× [(Z_α/2_ + Z_β_)^2^×σ^²^]/δ^²^.

An effect size of 8 = 0.76 was adopted, informed by prior SDL studies.^[11]^ Parameters included a two-tailed significance level of a = 0.05 (corresponding to 2,1 = 1.96) and a statistical power of 0.90 (corresponding to 2*g* = 1.28) with the standard deviation standardized as *o* = 1. This yielded an initial estimate of 37 participants per group. However, to account for potential attrition and ensure sufficient power to detect medium-to-large effects in the context of educational interventions, the sample size was increased to 45 participants per group, resulting in a total of 90 participants across both groups. This final sample size provides robust statistical power consistent with the study’s objectives.

### 2.2. Procedure

The study was conducted over 6 months, from July to December 2023, and incorporated multiple 4-week internship cycles involving distinct participant cohorts. Pre-intervention assessments, including baseline Critical Thinking Disposition Inventory-Chinese Version (CTDI-CV) and Self-Rating Scale for Self-Directed Learning (SRSSDL) scores, were administered on the first day of the rotation. The experimental group underwent a 4-week “Learning, Thinking & Conveying” training program combined with experiential learning. Post-intervention assessments, comprising written exams, clinical operation skills exams, CTDI-CV and SRSSDL scores, were conducted in the final week of each cycle.

### 2.3. Experimental group

**Figure 3.**
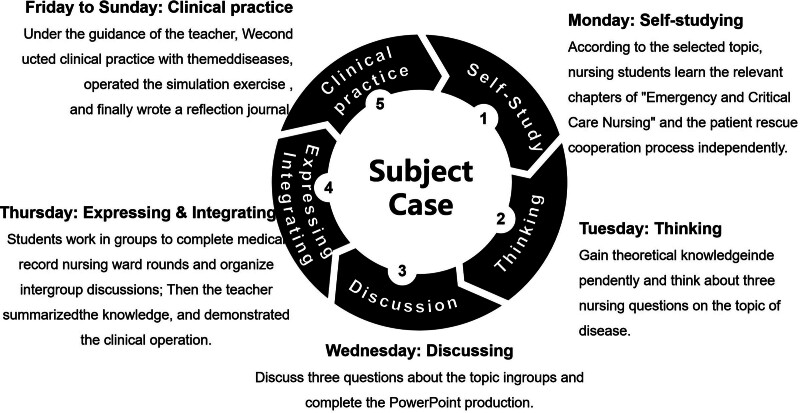
“Learning, Thinking & Conveying” training with experiential learning framework. Representation of the integration of “Learning, Thinking & Conveying” pedagogy with experiential learning within a training program tailored to meet hospital teaching requirements and the specific needs of clinical practice in the emergency department. The framework combines structured self-directed learning and critical thinking exercises with real-world application, designed to enhance the educational experience of nursing students through a hands-on approach. The elements are interwoven to support learning outcomes.

A training program was developed incorporating “Learning, Thinking & Conveying” training in conjunction with experiential learning of hospital teaching requirements and emergency department clinical practice needs. “Learning Thinking&Conveying” Training with Experiential Learning frame diagram is shown in Fig. 3. The program included 10 thematic medical records, covering emergency triage, sudden death, acute myocardial infarction, acute cerebral infarction, trauma, poisoning, and shock, with 3 randomly selected for each internship cycle. The program of “Learning Thinking & Conveying” training combined with experiential learning is shown in Table 1.

**Table 1:** Program of “Learning Thinking & Conveying” training combined with experiential learning.

Date/theme	Monday (self-studying: follow the textbook and process according to the topic case)	Tuesday (thinking: based on the theoretical knowledge and clinical practice, think about 3 issues related to the topic)	Wednesday (discussing: students in the same class will be grouped together and have group discussions online and offline)	Thursday (expressing: reporting the way of thinking in the form of nursing rounds) + (integrating: the teaching teacher made a summary speech, and carried out operation demonstration)	Friday to Sunday (combined with clinical practice for bedside instruction and teaching, one-on-one guidance supervision by teachers)
The first week	Learn textbook independently: Chapter on cardiac arrest and cardiopulmonary resuscitation; Emergency department rescue nursing cooperation process: Emergency nursing cooperation for sudden death patients	Think about the problem according to the theoretical knowledge related to sudden death: (1) What are the nursing procedures in the emergency department for the rescue of patients with sudden death? (2) What are the key steps in the emergency department’s nursing process to rescue sudden death patients? (3) What aspects should nurses pay attention to in emergency department to rescue sudden death patients?	(1) Discuss ideas and perspectives on 3 issues related to sudden death care (2) Summarize and integrate everyone’s views to reach a consensus (3) Take the group as a unit, under the guidance of the teaching teacher, complete the PPT production through teamwork	(1) Take the group as a unit to complete the nursing rounds of the theme case together (2) Organize discussions between groups (3) The teacher summarized and unified the discussion, and demonstrated the operation of CPR	Under the guidance of the teacher, we conducted clinical practice with themed diseases about sudden death, performed CPR exercises and rescue team coordination, and finally wrote a reflection journal.
Theme: the sudden death					
The second week	Learn textbook independently: Chapter on acute myocardial infarction; Emergency department rescue nursing cooperation process: Emergency nursing cooperation for acute myocardial infarction	Think about the problem according to the theoretical knowledge related to acute myocardial infarction: (1) What are the core steps of acute myocardial infarction care in the emergency department? (2) How is assessment and monitoring performed in the emergency department? What are the key considerations? (3) How can the nursing process in the emergency department be integrated with interventional treatment and complication management to ensure the best patient care outcomes?	(1) Discuss ideas and perspectives on 3 issues related to acute myocardial infarction (2) Summarize and integrate everyone’s views to reach a consensus (3) Take the group as a unit, under the guidance of the teaching teacher, complete the PPT production through teamwork	(1) Take the group as a unit to complete the nursing rounds of the theme case together (2) Organize discussions between groups (3) The teacher summarized and unified the discussion, and demonstrated the operation of 12-lead and 18-lead electrocardiograms	Under the guidance of the teacher, We conducted clinical practice with themed diseases about the acute myocardial infarction, performed 12-lead and 18-lead electrocardiogram exercises and rescue team coordination, and finally wrote a reflection journal.
Theme: acute myocardial infarction					
The third week	Learn textbook independently: Chapter on severe trauma; Emergency department rescue nursing cooperation process: Emergency nursing cooperation for severe trauma	Think about the problem according to the theoretical knowledge related to severe trauma: (1) How to conduct rapid assessment and prioritization? (2) How do you establish IV access, fluid resuscitation, and pain management? (3) How to monitor and collaborate on team communication?	(1) Discuss ideas and perspectives on 3 issues related to severe trauma (2) Summarize and integrate everyone’s views to reach a consensus (3) Take the group as a unit, under the guidance of the teaching teacher, complete the PPT production through teamwork	(1) Take the group as a unit to complete the nursing rounds of the theme case together (2) Organize discussions between groups (3) The teacher summarized and unified the discussion, and demonstrated the operation of limb trauma bandaging and fixation	Under the guidance of the teacher, We conducted clinical practice with themed diseases about the severe trauma, performed limb trauma bandaging and fixation exercises and rescue team coordination, and finally wrote a reflection journal.
Theme: trauma					
The fourth week	Under the guidance of the teacher, as an assistant to conduct clinical practice exercises				Evaluation of teaching and learning (summarize the learning experience of the emergency department, put forward opinions and suggestions)

Overview of teaching topics and content associated with the intervention at different periods: the program encompassed 10 thematic medical records, addressing areas such as emergency triage, sudden death, acute myocardial infarction, acute cerebral infarction, trauma, poisoning and shock. For each internship cycle, 3 topics were randomly selected, aligning with hospital teaching requirements and emergency department clinical practice needs.

### 2.4. Control group

Control nursing students received routine mentoring in the emergency department, as follows: (1) initial briefing from the chief instructor on hospital and department rules, overview of the environment, training and assessment processes. A weekly mentoring plan with final evaluation objectives and methods was presented. (2) Students received routine and specialized training, including CPR, gastric lavage and defibrillation, 4 times a week; (3) 8 bedside one-to-one teaching sessions on emergency triage, sudden death, acute myocardial infarction, acute cerebral infarction, trauma, poisoning, and shock; (4) teaching activity display in which student-led nursing rounds focused on typical acute and critical cases. (5) A final evaluation with a theoretical exam and practical assessment was conducted. Nursing students completed a critical thinking disposition questionnaire and an SDL evaluation questionnaire before leaving the department.

### 2.5. Instruments

#### 2.5.1. Demographic variable questionnaire

Demographic data thought to influence critical thinking,^[12,13]^ including gender, age, birthplace, student cadre, and voluntary selection of the nursing profession, were collated.

#### 2.5.2. Exam results

Final exam results on theoretical performance and clinical operation skills were compared between the 2 groups. The 90-minute theoretical exam (maximum score = 100 points) included single and multiple-choice objective questions plus short-answer and medical record analysis subjective questions. Experimental and control groups received the same exam paper with the same time limit and were evaluated by the same person. The clinical operation examination (maximum score = 100 points) consisted of a randomly selected operational item with uniform evaluation.

### 2.6. Critical thinking assessment

Critical thinking was assessed using the CTDI-CV which was adapted from the California Critical Thinking Disposition Inventory.^[14,15]^ The CTDI-CV has 7 subscales: truth-seeking, open-mindedness, analyticity, systematicity, critical thinking self-confidence, inquisitiveness, and cognitive maturitywith 70 items (10 per subscale) rated on a six-point Likert scale from “1” (completely agree) to “6” (completely disagree). Thirty items are phrased positively and 40 negatively. A higher score indicated a greater facility with critical thinking.

A subscale score of 40 or above indicated a facility in that particular aspect. A total score of 210 or lower reflected a lack of facility in critical thinking and 280 or above suggested good facility with 350 or higher being exceptional. Cronbach alpha for the CTDI-CV was reported as 0.90^[15]^ and confirmed in the current study (Cronbach alpha = 0.845).

### 2.7. SDL assessment

The SRSSDL is rated on a five-point Likert scale, ranging from “1” (never) to “5” (always) with 12 items per subscale, and was adapted from the self-rating scale of self-directed learning. Scores vary from 60 to 300 with higher scores indicating greater facility with SDL.^[16]^ Score categories were considered to be low level (60–140), moderate level (141–220) or high level (221–300). Cronbach alpha for SRSSDL was reported as 0.97 and content validity was 0.96.^[17]^ The Cronbach alpha coefficient of this questionnaire in the current study was 0.923.

### 2.8. Data collection methods

Nursing students completed the demographic variable questionnaire, CTDI-CV, and SRSSDL at the beginning of the rotation, following a uniform instructional speech. The CTDI-CV and SRSSDL were completed for the second time before the departmental rotation in the 4th week of the internship program, in addition to theoretical and clinical operation skills tests. Scales were collated immediately after the questionnaires were completed by an on-site researcher.

### 2.9. Ethical considerations

The study was conducted in compliance with the Declaration of Helsinki and received ethical approval from the Ethics Committee of Wuhan Central Hospital (approval number: WHZXKYL2023-077). All participants provided informed consent. Confidentiality of all personal data was maintained and data was used exclusively for this research.

### 2.10. Statistical analysis

Data analysis was performed using IBM Statistical Analysis Software v26.0 for Windows (IBM Corp., Armonk). Demographic characteristics of gender, age, place of birth, student cadre and voluntary choice of profession are presented as means and rates and were compared using independent sample *t* tests and *χ*² tests. SDL and critical thinking before and after training were assessed with paired sample *t* tests and independent sample *t* tests. Differences in theoretical performance and clinical operation skills were evaluated using independent *t* tests. Data were assessed for normality using the Shapiro–Wilk test prior to conducting linear regression analyses, homoscedasticity via scatterplots of residuals and multicollinearity by examining variance inflation factors, with all variance inflation factor values below 5 indicating no significant collinearity. Outcome indicators were correlated with demographic data using multivariate linear regression analysis, controlling for confounding covariates of gender, age, place of birth, student cadre and voluntary choice of profession. Covariates were selected based on prior studies demonstrating their influence on learning outcomes in nursing education^[18,19]^ and theoretical reasoning linking these factors to motivation and skill acquisition. Univariate and multivariate linear regression analyses were conducted to assess factors influencing outcome indicators with *P* < .05 indicating statistical significance.

## 3. Results

### 3.1. Baseline characteristics

No significant differences were observed in demographic characteristics between the control and experimental groups. Demographic characteristics of experimental and control groups are shown in Table 2.

**Table 2:** Demographic characteristics of experimental (n = 45) and control (n = 45) groups.

Characteristics		Experimental group (n = 45), n(%)	Control group (n = 45), n(%)	*X* ^*2*^ */t*	*P*
Gender				0.090	.764
	Female	38 (84.4)	39 (86.7)		
	Male	7 (15.6)	6 (13.3)		
Age (years), M ± SD		20.07 ± 0.915	20.16 ± 0.903	‐0.230	.818
Place of birth				0.045	.832
	City or town	19 (42.2)	20 (44.4)		
	Countryside	26 (57.8)	25 (55.6)		
Student cadre				0.227	.634
	Yes	13 (28.9)	11 (24.4)		
	No	32 (71.1)	34 (75.6)		
Choose nursing profession voluntarily				0.385	.535
	Yes	7 (15.6)	5 (5.6)		
	No	38 (84.4)	40 (88.9)		

Baseline demographic characteristics of participants in experimental and control groups. No significant differences were observed, indicating comparability.

M = mean, SD = standard deviation.

### 3.2. Exam results

Theoretical performance was significantly higher among nursing students in the experimental group compared with controls but no significant difference was found for clinical operation skills. Theoretical and practical exam results before and after the study are given in Table 3. An impact of “Learning, Thinking & Conveying” Training with Experiential Learning on theoretical exam results (β = 0.731, *P* < .001) was shown by univariate linear regression analysis and this relationship remained after mutual adjustment by multivariate linear regression (Table 4). No association was found for gender, age, place of birth, student cadre or voluntary choice of nursing profession. Students who received “Learning, Thinking & Conveying” Training with Experiential Learning thus had higher theoretical exam results than controls (β = 0.727, *P* < .001). Neither univariate linear regression analysis nor mutual adjustment through multivariate linear regression revealed any category of factors that were significantly associated with practical exam results (Table 5).

**Table 3:** Theoretical and practical exam results before and after the study (n = 90).

Item	Experimental group(n = 45) mean ± SD	Control group(n = 45) mean ± SD	*t*	*P*
Theoretical examination	90.69 ± 3.895	81.73 ± 4.545	10.037	<.001
Clinical operation skills examination	92.64 ± 3.255	92.80 ± 3.087	-0.233	.817

Theoretical knowledge and clinical operation skills exam results for nursing students in experimental and control groups. The experimental group demonstrated significantly higher theoretical performance following training compared with controls. There was no significant difference in clinical operation skills between the 2 groups.

M = mean, SD = standard deviation.

**Table 4:** Factors associated with the theoretical exam results according to linear regression analysis (n = 90).

Characteristics		Univariate model β (95% CI)	*P* value	Multivariate model β (95% CI)	*P* value
Gender					
	Male*	–†	–	–	–
	Female	0.099(‐1.944 to 5.407)	.352	0.036(‐1.460 to 2.706)	.553
Age (years)		‐.008(‐1.499 to 1.386)	.938	0.041(‐0.500 to 1.065)	.475
Place of birth					
	City or town*	–	–	–	–
	Countryside	0.074(‐1.698 to 3.529)	.488	0.011(‐1.315 to 1.584)	.854
Student cadre					
	Yes*	–	–	–	–
	No	‐.110(-4.449 to 1.388)	.300	‐0.016(‐1.890 to 1.445)	.791
Choose nursing profession voluntarily					
	Yes*	–	–	–	–
	No	‐.045(‐4.630 to 3.002)	.673	0.020(‐1.788 to 2.514)	.738
“Learning, Thinking & Conveying” training with experiential learning					
	Yes*	–	–	–	–
	No	0.731(7.182 to 10.729)	.000	0.727(7.510 to 10.314)	.000

Univariate and multivariate linear regression analyses for factors influencing theoretical exam results. A significant positive impact of “Learning, Thinking & Conveying” training with experiential learning was demonstrated (univariate: β = 0.731, *P* < .001; multivariate: β = 0.727, *P* < .001). The positive effect persisted after adjustment for other factors. No significant associations were found for gender, age, place of birth, student cadre status or voluntary choice of the nursing profession, indicating that the training was the primary differentiator in performance.

* Reference group.

† Not applicable.

**Table 5:** Factors associated with the practical exam results according to linear regression analysis (n = 90).

Characteristics		Univariate model β (95% CI)	*P* value	Multivariate model β (95% CI)	*P* value
Gender					
	Male*	–†	–	–	–
	Female	‐0.095 (‐2.727 to 1.038)	.375	‐0.105 (‐2.920 to 1.052)	.352
Age(yr)		‐0.158 (‐1.281 to 0.177)	.136	‐0.145 (‐1.254 to 0.241)	.181
Place of birth					
	City or town*	–	–	–	–
	Countryside	0.066 (‐.924 to 1.753)	.540	0.034 (‐1.143 to 1.580)	.751
Student cadre					
	Yes*	–	–	–	–
	No	‐0.125 (‐2.381 to 0.601)	.239	‐0.143 (‐2.599 to 0.571)	.207
Choose nursing profession voluntarily					
	Yes*	–	–	–	–
	No	‐0.045 (‐2.370 to 1.537)	.673	0.009 (‐1.974 to 2.135)	.938
“Learning, Thinking & Conveying” Training with Experiential Learning					
	Yes*	–	–	–	–
	No	‐0.025 (‐1.485 to 1.173)	.817	‐0.036 (‐1.565 to 1.113)	.738

Univariate and multivariate linear regression analyses evaluate factors potentially associated with practical exam results. No significant associations between practical exam results and “Learning, Thinking & Conveying” training, gender, age, place of birth, student cadre and voluntary choice of the nursing profession were found.

* Reference group.

† Not applicable.

### 3.3. SDL

Dimensions and total scores of the SRSSDL before and after the study are shown in Table 6. No differences in SRSSDL scores were found before training but the experimental group had higher scores in each dimension and total after training than controls. SRSSDL results were higher for the experimental group following internship than before but there was no effect of training for the control group. Univariate linear regression analysis showed that “Learning, Thinking & Conveying” Training with Experiential Learning (β = 0.284, *P* = .007) had an impact on total SRSSDL scores which persisted after mutual adjustment by multivariate linear regression but no association was found for gender, age, place of birth, student cadre or voluntary choice of nursing profession (Table 7). Those who received “Learning, Thinking & Conveying” Training with Experiential Learning thus had higher total SRSSDL scores after training (β = 0.271, *P* = .011).

**Table 6:** Dimensions and total SRSSDL scores before and after training (n = 90).

Items	Experimental group (n = 45)		Within the group		Control group (n = 45)		Within the group		Between groups (before training)		Between groups (after training)	
	Before training mean ± SD	After training mean ± SD	*t*	*P*	Before training mean ± SD	After training mean ± SD	*t*	*P*	* *t*	* *P*	* *t*	* *P*
Learning awareness	38.07 ± 6.69	40.84 ± 5.92	‐7.187	<.001	37.82 ± 5.93	37.67 ± 5.54	0.654	.516	0.184	.855	2.629	.01
Learning strategies	39.47 ± 5.60	42.42 ± 6.01	‐10.533	<.001	39.40 ± 5.75	39.82 ± 5.04	-1.642	.108	0.056	.956	2.051	.043
Learning activities	39.53 ± 5.31	41.89 ± 5.61	‐10.465	<.001	38.82 ± 5.04	39.04 ± 5.01	-0.896	.375	0.652	.516	2.520	.014
Learning evaluation	40.22 ± 6.04	42.60 ± 6.15	‐11.505	<.001	40.40 ± 5.17	40.09 ± 5.22	0.853	.399	-0.150	.811	2.088	.040
Interpersonal skills	41.49 ± 6.30	44.78 ± 5.89	‐14.403	<.001	41.33 ± 5.51	41.47 ± 5.30	-0.453	.652	0.125	.901	2.805	.006
Total score	198.78 ± 27.00	212.53 ± 25.80	‐18.709	<.001	179.78 ± 24.00	198.09 ± 23.37	-0.422	.675	0.186	.853	2.783	.007

Dimensions and total Self-Directed Learning Readiness Scale for Nursing Education (SRSSDL) scores before and after training. No significant differences in SRSSDL scores were observed between experimental and control groups at baseline. The experimental group exhibited higher posttraining scores across all dimensions and in total compared with controls. Additionally, the experimental group showed increased SRSSDL scores following their internship whereas no change was found for controls.

M = mean, SD = standard deviation.

* The independent-sample *t* test.

**Table 7:** Factors associated with the number of total SRSSDL scores after training according to linear regression analysis (n = 90).

Characteristics		Univariate model β (95% CI)	*P* value	Multivariate model β (95% CI)	*P* value
Gender					
	Male*	–†	–	–	–
	Female	0.078(‐9.594 to 20.914)	.463	0.057(‐11.506 to 19.689)	.603
Age (years)		‐0.053(‐7.452 to 4.486)	.623	‐0.038(‐6.952 to 4.789)	.715
Place of birth					
	City or town*	–	–	–	–
	Countryside	0.036(-8.986 to 12.709)	.734	0.020(-9.686 to 11.695)	.852
Student cadre					
	Yes*	–	–	–	–
	No	-0.165(-21.459 to 2.535)	.121	-0.132(-20.009 to 4.889)	.231
Choose nursing profession voluntarily					
	Yes*	–	–	–	–
	No	‐0.074(‐21.287 to 10.274)	.490	‐0.034(‐18.707 to 13.561)	.752
“Learning, Thinking & Conveying” Training with Experiential Learning					
	Yes*	–	–	–	–
	No	0.284(4.131 to 24.758)	.007	0.271(3.267 to 24.302)	.011

Univariate and multivariate linear regression analyses of factors associated with total SRSSDL scores following training. “Learning, Thinking & Conveying” training with experiential learning significantly improved total SRSSDL scores (univariate: β = 0.284, p = 0.007; multivariate: β = 0.271, p = 0.011). This effect remained significant after adjustment for other variables. No significant associations were found for gender, age, place of birth, student cadre status or voluntary choice of the nursing profession, indicating that the nature of training was the influential factor.

* Reference group.

† Not applicable.

### 3.4. Critical thinking

No difference in pretraining CTDI-CV scores was found between the experimental and control groups but the dimensions of truth-seeking, open-mindedness, analyticity, critical thinking self-confidence, inquisitiveness and total scores were significantly different between the 2 groups following the internship. Systematicity and cognitive maturity scores were higher but not significantly different in the experimental group. No significant differences were observed in total CTDI-CV scores or any subscales for the control group before and after training. Dimensions and total CDTI-CV scores before and after training are shown in Table 8. Univariate linear regression analysis showed that “Learning, Thinking & Conveying” training with experiential learning (β = 0.284, *P* = .007) was associated with higher CDTI-CV scores and this effect persisted after mutual adjustment by multivariate linear regression (Table 9). No effect of gender, age, place of birth, student cadre or voluntary choice of nursing profession was found. Recipients of “Learning, Thinking & Conveying” training with experiential learning thus had higher total CDTI-CV scores after training (β = 0.268, *P* = .012).

**Table 8:** Dimensions and total CDTI-CV scores before and after training (N = 90).

Items	Experimental group (n = 45)		Within the group		Control group (n = 45)		Within the group		Between groups (before training)		Between groups (after training)	
	Before training mean ± SD	After training mean ± SD	*t*	*P*	Before training mean ± SD	After training mean ± SD	*t*	*P*	* *t*	* *P*	* *t*	* *P*
Truth seeking	38.47 ± 4.475	41.67 ± 5.427	‐8.260	<.001	38.87 ± 3.715	39.22 ± 4.379	-0.910	.368	-0.461	.646	2.351	.021
Open-mindedness	43.89 ± 3.961	44.98 ± 4.109	‐3.742	.001	43.00 ± 3.219	42.64 ± 3.311	1.012	.317	1.168	.246	2.966	.004
Analyticity	43.76 ± 3.588	45.53 ± 3.751	‐6.298	<.001	43.38 ± 3.839	43.13 ± 3.709	0.604	.549	0.482	.631	3.052	.003
Systematicity	38.00 ± 2.714	38.67 ± 3.873	‐1.907	.063	38.38 ± 2.782	38.62 ± 3.193	-0.659	.513	-0.652	.516	0.059	.953
Critical thinking self-confidence	40.09 ± 4.476	42.98 ± 4.120	‐8.778	<.001	40.24 ± 4.344	40.73 ± 4.092	-1.000	.323	-0.167	.868	2.593	.011
Inquisitiveness	42.27 ± 3.869	44.02 ± 4.251	‐6.170	<.001	42.18 ± 3.792	41.89 ± 3.485	0.704	.485	0.110	.913	2.604	.011
Cognitive maturity	42.53 ± 6.189	42.69 ± 5.534	‐0.569	.572	42.44 ± 4.765	42.16 ± 4.311	0.772	.444	0.076	.939	0.510	.611
Total score	289.00 ± 21.635	300.53 ± 22.358	‐12.955	<.001	288.49 ± 22.012	288.40 ± 18.878	0.060	.952	0.111	.912	2.782	.007

Dimensions and total scores of the California Critical Thinking Disposition Inventory-Chinese Version (CTDI-CV) before and after training for experimental and control groups. No differences were observed between the 2 groups at baseline. However, the experimental group showed significant increases in the dimensions of truth-seeking, open-mindedness, analyticity, critical thinking self-confidence, inquisitiveness and in total CTDI-CV post-training scores compared with controls. Scores for systematicity and cognitive maturity were nonsignificantly higher in the experimental group. The control group showed no significant changes in total CTDI-CV scores or any subscales before and after training.

M = mean, SD = standard deviation.

* The independent-sample *t* test.

**Table 9:** Factors associated with the number of total CDTI-CV scores after training according to linear regression analysis (n = 90).

Characteristics		Univariate model β (95% CI)	*P* value	Multivariate model β (95% CI)	*P* value
Gender					
	Male*	–†	–	–	–
	Female	0.080 (‐7.970 to 17.668)	.454	0.085 (‐7.914 to 18.212)	.435
Age (years)		‐0.032 (‐5.785 to 4.257)	.763	‐.015 (‐5.277 to 4.556)	.884
Place of birth					
	City or town*	–	–	–	–
	Countryside	0.020 (‐9.686 to 11.695)	.852	.041 (‐7.209 to 10.698)	.699
Student cadre					
	Yes*	–	–	–	–
	No	‐0.129 (‐16.376 to 3.899)	.225	‐.070 (‐13.808 to 7.043)	.521
Choose nursing profession voluntarily					
	Yes*	–	–	–	–
	No	‐0.154 (‐22.795 to 3.487)	.148	‐0.131 (‐21.767 to 5.258)	.228
“Learning, Thinking & Conveying” Training with Experiential Learning					
	Yes*	–	–	–	–
	No	0.285 (3.464 to 20.802)	.008	.268 (2.622 to 20.239)	.012

Linear regression analyses of factors influencing total CTDI-CV scores following training. Univariate analysis showed that “Learning, Thinking & Conveying” training with experiential learning was associated with increased CTDI-CV scores (β = 0.284, *P* = .007). This association remained significant after adjustments in multivariate analysis (β = 0.268, *P* = .012). The analysis found no significant effects based on gender, age, place of birth, student cadre status or voluntary choice of the nursing profession. Thus, the nature of training was the factor influencing critical thinking skills.

* Reference group.

† Not applicable.

## 4. Discussion

“Learning, Thinking & Conveying” training, combined with experiential learning provided a constructive opportunity for undergraduate nursing students to improve their performance by increasing their SDL and critical thinking. Theoretical knowledge was enhanced and was significantly greater than for controls but no impact on clinical operation skills was found. We consider that these results demonstrate the superiority of “Learning, Thinking & Conveying” with experiential learning over traditional educational methods. The focus on self-learning, finding useful information, thinking about related problems, group discussion, and nursing rounds may have contributed to the training enhancement. Traditional teaching methods of independent learning with teacher-led instruction and limited feedback may not facilitate as great a depth of understanding.

Clinical operation skills did not appear to be enhanced by the current teaching approach, perhaps because the training was similar for both groups to ensure quality control. All students were required to master operational skills to a set standard. The lack of impact of “Learning, Thinking & Conveying” training on clinical operations skills may indicate the need to augment the practical, hands-on component of the nursing course to extend the benefits to clinical proficiency.

“Learning, Thinking & Conveying” with experiential learning also improved SDL facility, a key goal of nursing education.^[20]^ Nursing staff are expected to undertake continuous knowledge updating through SDL^[21]^ and SDL is considered to enhance knowledge, self-confidence, and satisfaction in nursing students.^[22]^ The current experimental cohort had higher SDL total and subscale scores following training compared with controls. The teacher-guided approach of traditional teaching methods may limit students’ initiative but “Learning, Thinking & Conveying” training motivates students to be proactive and critically apply theoretical knowledge to teacher-raised problems.

“Learning, Thinking & Conveying” with experiential learning, transcends traditional teaching modes, integrating knowledge instruction, and skill development. The current study emphasizes the importance of timely feedback for learning outcomes, aiding nursing students in adjusting their approaches and transitioning from being educated to engaging in clinical practice. Students become more focused, organized and diligent in clinical practice.

Sun et al^[23]^ found the combination of the flipped classroom combined with virtual simulation platform used in blended teaching to enhance SDL, as did participatory learning.^[24]^ Experiential teaching in combination with case teaching also stimulated SDL, interest, and enthusiasm.^[25]^ Lifelong learning is essential for nursing professionals and clinical teaching should focus on stimulating students’ enthusiasm and independent learning skills. “Learning, Thinking & Conveying” training relied on both independent and group learning styles with the involvement of continuous mentor support to develop SDL skills.^[10]^

Kolb experiential learning theory states that adult higher education is learner-centered, self-motivated, and self-instructed.^[26]^ Students acquire and update knowledge through experiential learning. Experiential teaching was found to improve SDL and clinical decision-making.^[27]^ Students learn and reflect independently, share views and achieve the satisfaction of finding and solving problems, fostering a continuous desire to learn.^[10]^

“Learning, Thinking & Conveying” training with experiential learning helps to improve critical thinking which nurses must cultivate for job satisfaction and enhanced patient outcomes.^[6]^ Pretraining critical thinking scores were 289.00 ± 21.635 for the experimental cohort and 288.49 ± 22.012 for controls, reflecting well-developed critical thinking, consistent with previous findings.^[28,29]^ However, overall mean scores were lower in the present study than in that of Boso,^[6]^ possibly indicating cultural differences.

Truth-seeking, open-mindedness, analyticity, critical thinking self-confidence, inquisitiveness and total critical thinking scores were all higher for the experimental than for the control group following training. The “Learning, Thinking & Conveying” training facilitated problem-based and student-centered learning allowing the acquisition and application of theoretical knowledge. A meta-analysis suggested that dedicating more time to active, meaningful learning and thinking processes was beneficial^[30]^ and problem-based learning coupled with active learning improved critical thinking in nursing students regarding Basic Life Support.^[31]^ Pre-class tasks encouraged enhanced information collection, analysis and problem-solving skills, indicating that sufficient time should be dedicated to experiential learning in health services.

Experiential teaching aids the analysis of patient needs and the overcoming of clinical communication barriers. Scenario simulation practice may pose questions such as, “If the patient suddenly has difficulty breathing at this time, how should you deal with such an emergency?” to enhance the alertness of the student’s responses and encourage critical thinking. Experiential teaching improves nursing students’ humanistic qualities, communication, critical thinking and SDL,^[32]^ promoting the connection of theoretical knowledge with real nursing practice.^[6]^ Thus, systematicity was reinforced among the current cohort. Systematicity and cognitive maturity scores were higher in the experimental group but differences did not reach statistical significance. Systematicity reflects an organized and methodical approach to problem-solving and cognitive maturity indicates the perception of problems as complex rather than black and white.^[14]^ Both traits were relatively stable and the current training duration may have been insufficient to produce a measurable influence.

The study was conducted in the emergency department of a tertiary care hospital in Wuhan, Hubei Province which takes nursing students from various medical schools. Clinical scenario simulations were incorporated into the training of the experimental group to assess and provide prompt feedback on critical thinking and SDL. Mentors evaluated students’ mastery of knowledge and adapted teaching strategies accordingly. It is noteworthy that covariates of age, gender and voluntary choice of nursing profession did not impact study outcomes. The current cohort was overwhelmingly female, aged between 19 and 22 years and had voluntarily chosen their career. It is possible that a cohort with a different demographic would have produced different outcomes. However, we consider that the cohort reported in the study is broadly representative of nursing recruitment throughout China, giving the findings general applicability.

The current review has the strength of assessing “Learning, Thinking & Conveying” training with experiential learning for undergraduate nursing students for the first time. We also acknowledge some limitations. Firstly, bimonthly enrollment was employed to minimize subject selection bias since nurses arrived in groups. A randomized clinical trial with rigorous control of heterogeneity factors may have been preferable. In addition, the sample size, although carefully chosen to ensure appropriate statistical analysis, was small. Future studies with a larger cohort of student nurses would validate the current findings. Secondly, the experimental period was of short duration, raising concerns about the generalizability of results. Further studies that extend the scope beyond the 4-week training period would address this deficiency. Thirdly, the analysis of prior academic achievement, personal motivation and individual learning strategies fell outside the scope of the current experimental approach and could not be evaluated. These factors may influence exam performance and could provide additional insights into the observed outcomes, representing an opportunity for future studies to expand on the current findings. Lastly, student questionnaires involved self-assessment which may have introduced a degree of subjectivity, although this approach is commonly used in research. Future research may assess the long-term impact of learning methods on nursing practice with face-to-face interviews during a follow-up period to give deeper insights.

## 5. Conclusion

Blended teaching methods enhance self-directed learning and critical thinking among undergraduate nursing students. “Learning, Thinking & Conveying” training with experiential learning is a highly effective educational approach.

## Author contributions

**Conceptualization:** Di Liu.

**Data curation:** Di Liu.

**Formal analysis:** Ying Li.

**Investigation:** Ying Li, Shuang Cheng, Di Liu, Wei Jiang.

**Methodology:** Ying Li, Shuang Cheng, Di Liu, Wei Jiang.

**Resources:** Di Liu.

**Software:** Ying Li, Shuang Cheng.

**Supervision:** Ying Li, Wei Jiang.

**Validation:** Di Liu.

**Writing – original draft:** Ying Li, Shuang Cheng, Di Liu, Wei Jiang.

**Writing – review & editing:** Ying Li, Shuang Cheng.

## References

[1] González-García M Lana A Zurrón-Madera P Valcárcel-Álvarez Y Fernández-Feito A. Nursing students’ experiences of clinical practices in emergency and intensive care units. Int J Environ Res Public Health. 2020;17:5686. 32781646 10.3390/ijerph17165686 PMC7459869

[2] Wu ZX. Application of the “Xue Si Da” teaching method from the perspective of autonomous learning. Basic Educ Res. 2021;15:35–37.

[3] Zhang H. Xuesida I: Zhang Huicheng’s Flipped Teaching Practice. Beijing Times Chinese Bookstore; 2019.

[4] Cheng YC Huang LC Yang CH Chang H-C. Experiential learning program to strengthen self-reflection and critical thinking in freshmen nursing students during COVID-19: a quasi-experimental study. Int J Environ Res Public Health. 2020;17:5442. 32731648 10.3390/ijerph17155442 PMC7432080

[5] Halpern DF. Teaching for critical thinking: Helping college students develop the skills and dispositions of a critical thinker. New Directions Teach Learn. 1999;1999:69–74.

[6] Boso CM van der Merwe AS Gross J. Critical thinking disposition of nursing students: a quantitative investigation. Nurse Educ Pract. 2021;55:103167. 34358855 10.1016/j.nepr.2021.103167

[7] Chan ZC. A systematic review of critical thinking in nursing education. Nurse Educ Today. 2013;33:236–40. 23394977 10.1016/j.nedt.2013.01.007

[8] Falcó-Pegueroles A Rodríguez-Martín D Ramos-Pozón S Zuriguel-Pérez E. Critical thinking in nursing clinical practice, education, and research: from attitudes to virtue. Nurs Philosophy. 2021;22:e12332. 10.1111/nup.12332 33029860

[9] Cadorin L Bressan V Palese A. Instruments evaluating the self-directed learning abilities among nursing students and nurses: a systematic review of psychometric properties. BMC Med Educ. 2017;17:229. 29178924 10.1186/s12909-017-1072-3 PMC5702155

[10] Wong FMF Tang ACY Cheng WLS. Factors associated with self-directed learning among undergraduate nursing students: a systematic review. Nurse Educ Today. 2021;104:104998. 34139583 10.1016/j.nedt.2021.104998

[11] Xiong Y Xu YL Hu LZ. Effect of online teaching platform in clinical nursing practice of gastroenterology. J Nurs (China). 2017;24:27–32.

[12] Mahmoud AS Mohamed HA. Critical thinking disposition among nurses working in public hospitals at Port-Said Governorate. Int J Nurs Sci. 2017;4:128–34. 31406732 10.1016/j.ijnss.2017.02.006 PMC6626108

[13] Wu HL Lu DF Tsee PK. Critical thinking disposition and influencing factors among new graduate nurses. J Continuing Educ Nurs. 2023;54:233–40. 10.3928/00220124-20230405-08 37134318

[14] Facione PA Facione NC Giancarlo CAF. The California Critical Thinking Disposition Inventory (CCTDI): Test Administration Manual. The California Academic Press; 1992.

[15] Peng MC Wang GC Chen JL. Validity and reliability of the Chinese critical thinking disposition inventory. Chin J Nurs. 2004;39:644–7.

[16] Williamson SN. Development of a self-rating scale of self-directed learning. Nurse Res. 2007;14:66–83. 17315780 10.7748/nr2007.01.14.2.66.c6022

[17] Shen WQ Hu Y. Reliability and validity of the Chinese version of the self-directed learning rating scale. Chin J Nurs. 2011;46:1211–3.

[18] Ten Hoeve Y Castelein S Jansen WS Jansen GJ Roodbol PF. Nursing students’ changing orientation and attitudes towards nursing during education: a two-year longitudinal study. Nurse Educ Today. 2017;48:19–24. 27697678 10.1016/j.nedt.2016.09.009

[19] Tiwari A Avery A Lai P. Critical thinking disposition of Hong Kong Chinese and Australian nursing students. J Adv Nurs. 2003;44:298–307. 14641400 10.1046/j.1365-2648.2003.02805.x

[20] Chen KS Monrouxe L Lu YH. Academic outcomes of flipped classroom learning: a meta-analysis. Med Educ. 2018;52:910–24. 29943399 10.1111/medu.13616 PMC6120558

[21] Cheng SF Kuo CL Lin KC Lee-Hsieh J. Development and preliminary testing of a self-rating instrument to measure self-directed learning ability of nursing students. Int J Nurs Stud. 2010;47:1152–8. 20223455 10.1016/j.ijnurstu.2010.02.002

[22] Park H Cho H. Effects of a self-directed clinical practicum on self-confidence and satisfaction with clinical practicum among South Korean nursing students: a mixed-methods study. Int J Environ Res Public Health. 2022;19:5231. 35564625 10.3390/ijerph19095231 PMC9104650

[23] Sun L Liu D Lian J Yang M. Application of flipped classroom combined with virtual simulation platform in clinical biochemistry practical course. BMC Med Educ. 2023;23:771. 37845661 10.1186/s12909-023-04735-x PMC10577961

[24] Cook JA Shore SE Burke-Miller JK. Participatory action research to establish self-directed care for mental health recovery in Texas. Psychiatr Rehabil J. 2010;34:137–44. 20952367 10.2975/34.2.2010.137.144

[25] Surapaneni KM. METAPAD” (METAbolic PAthways Decoded) – a gaming innovation to ease the complexity of metabolic pathways by promoting self-directed, active, participatory learning in small groups. BMC Med Educ. 2023;23:608. 37626407 10.1186/s12909-023-04587-5 PMC10464076

[26] Kolb DA. Experiential Learning: Experience as the Source of Learning and Development. Prentice Hall; 1984:16–17.

[27] Zhang QY Ma XS Cui QG Xiao HY Jin X. Effectiveness of experiential teaching method on the development of nursing students’ skill competence: a systematic review and meta-analysis. Front Nurs. 2020;7:70–9.

[28] Ju C Jin K. Effects of flipped learning on the critical thinking disposition, academic achievement, and academic self-efficacy of nursing students: a mixed methods study. J Korean Acad Soc Nurs Educ. 2020;26:25–35.

[29] Zhang C Fan H Xia J Guo H Jiang X Yan Y. The effects of reflective training on the disposition of critical thinking for nursing students in China: a controlled trial. Asian Nurs Res. 2017;11:194–200. 10.1016/j.anr.2017.07.002 28991600

[30] Fernández-Basanta S Picallo-García L Movilla-Fernández MJ. Cultivating learning in vitro: a meta-ethnography of learning experiences of nursing students regarding high-fidelity simulation. J Clin Nurs. 2023;32:2056–72. 35233846 10.1111/jocn.16269

[31] Carbogim FDC Barbosa ACS de Oliveira LB. Educational intervention to improve critical thinking for undergraduate nursing students: a randomized clinical trial. Nurse Educ Pract. 2018;33:121–6. 30293053 10.1016/j.nepr.2018.10.001

[32] Park M Jeong M Lee M Cullen L. Web-based experiential learning strategies to enhance the evidence-based-practice competence of undergraduate nursing students. Nurse Educ Today. 2020;91:104466. 32454317 10.1016/j.nedt.2020.104466

